# Current and Emerging Approaches to Engineer Antibacterial and Antifouling Electrospun Nanofibers

**DOI:** 10.3390/ma11071059

**Published:** 2018-06-22

**Authors:** Irene S. Kurtz, Jessica D. Schiffman

**Affiliations:** Department of Chemical Engineering, University of Massachusetts Amherst, Amherst, MA 01003-9303, USA; ikurtz@umass.edu

**Keywords:** anti-bio adhesion, antifouling, antibacterial, bio-interfaces, hierarchical nanofibers, nanofibers electrospinning, electrospun materials, self-cleaning fabrics

## Abstract

From ship hulls to bandages, biological fouling is a ubiquitous problem that impacts a wide range of industries and requires complex engineered solutions. Eliciting materials to have antibacterial or antifouling properties describes two main approaches to delay biofouling by killing or repelling bacteria, respectively. In this review article, we discuss how electrospun nanofiber mats are blank canvases that can be tailored to have controlled interactions with biologics, which would improve the design of intelligent conformal coatings or freestanding meshes that deliver targeted antimicrobials or cause bacteria to slip off surfaces. Firstly, we will briefly discuss the established and emerging technologies for addressing biofouling through antibacterial and antifouling surface engineering, and then highlight the recent advances in incorporating these strategies into electrospun nanofibers. These strategies highlight the potential for engineering electrospun nanofibers to solicit specific microbial responses for human health and environmental applications.

## 1. Introduction

Contamination by microorganisms detrimentally impacts a wide-range of industries, including medicine, separations, and marine vessels [1,2,3]. For instance, biofouling on the hulls of ships increases their roughness, consequently increasing their hydrodynamic drag as the vessel moves through the water [4]. The higher drag leads to a greater fuel consumption, exorbitant hull cleaning/repainting requirements, and associated environmental compliance measures. New materials or surface coatings, like electrospun nanofiber mats that can be engineered to delay or potentially prevent the attachment of microorganisms, are desperately needed. Materials can be engineered to be antibacterial and/or antifouling, which, although they are related concepts, refer to distinct phenomena in microbiology.

Antibacterial surfaces disrupt bacterial cells, causing cell death. In general, antibacterial surfaces alter the architecture of platforms to reduce or possibly eliminate the adherence of microorganisms and the subsequent formation of biofilms [5]. As a key aspect of modern medicine, antibacterial agents have greatly reduced the number of mortalities that result from bacterial infections. Unfortunately, globalization and a decline in antibiotic discovery correspond with the continued rise of antibiotic-resistant bacteria. Each year in the United States, antibiotic resistance directly results in 23,000 deaths, and it is predicted that 10 million deaths per year will occur worldwide by 2050 [6,7]. Although more deaths still occur worldwide because of lack of access to antibacterial agents than to antibiotic-resistant bacteria, the number of β-lactamases that have been identified since 1990 has increased ten-fold, thus demonstrating the increasing problems that are associated with antibiotic-resistant bacteria [8,9,10].

Broad-spectrum antibacterial agents are both inefficient and contribute to antibiotic resistance through their methods for inactivating bacteria. The efficacy of β-lactam antibiotics, one of the most established and widely used antibiotic therapies, have long been thought to arise from inactivating penicillin-binding proteins, which are interfering with the bacterial cell wall assembly. Recent evidence reveals a much more complex mode of action with β-lactams disorganizing bacterial cell-wall synthesis machinery in a multifaceted process that depletes cellular resources and causes cell-wall degradation [11]. The discovery of more complicated processes that are involved with highly-established broad-spectrum antibiotics illustrates the necessity for fundamental scientific research and more targeted antibacterials.

The alternatives for combatting bacteria that do not contribute to antibiotic resistance, specifically more targeted antibacterial agents or antifouling materials, are needed to replace the commonly employed broad-spectrum antibacterials [12]. Fundamental scientific research must be employed to identify genus- and species-specific pathways to target, replacing current methods that attack broad areas such as cell-wall synthesis, translation, and DNA synthesis [13]. Through rational drug design, more targeted antibacterials can attack highly-conserved active sites in bacteria, making antibacterials more specific and effective [14].

Similarly, engineering antifouling materials, which address biofouling without killing the bacteria, may be an effective way to avoid the development of resistance genes. Antifouling surfaces resist or prevent cellular attachment, because the microorganisms sense an unfavorable chemistry or surface topography [15]. The incorporation of a non-toxic polymer, such as poly(ethylene glycol) (PEG), is a common means of inhibiting bacterial adhesion, since the lack of cell binding leads to a lack of recognition by the immune system [16,17]. The use of surface chemistry and topography, for example, transforming a surface to be superhydrophobic (e.g., lotus leaf) or to have a engineered topography (e.g., shark-skin [18]), are other common methods of adding antifouling properties to surfaces [19,20,21].

While a large range of possibilities remain for the development of antibacterial and antifouling technologies, a universal platform that economically imparts these properties to a wide range of surfaces is needed. Compared to other materials, the electrospun nanofiber mats exhibit unrivaled properties, including a high surface to volume ratio, which leads to a high permeability and porosity. The electrospun nanofiber mats that are either functionalized or incorporate alternative (i.e., ‘greener’) antibacterial agents could serve as conformal surface coatings that eliminate the spread of detrimental microorganisms. In this review article, we present general strategies and emerging technologies towards tailoring antibacterial and antifouling electrospun nanofibers.

## 2. Brief Background on the Electrospinning Process and General Strategies to Tailor Antibacterial and Antifouling Nanofiber Mats

The modern electrospinning process is well-established, drawing foundational knowledge from early theories on electrodynamics [22,23]. Significant progress has been documented in patents [24,25,26,27,28], in terms of dictating the basic requirements to build an electrospinning apparatus that can produce small quantities of nanofibers for basic research applications, as well as large quantities of nanofibers that are relevant for industrial products [29]. Because comprehensive review articles [30,31] have discussed theoretical models about electrospinning, how to produce nanofibers, how to chemically and physically characterize the produced mats, and current applications of nanofibers, here, we provide only a brief overview of the process so as to provide context.

**Figure 1:**
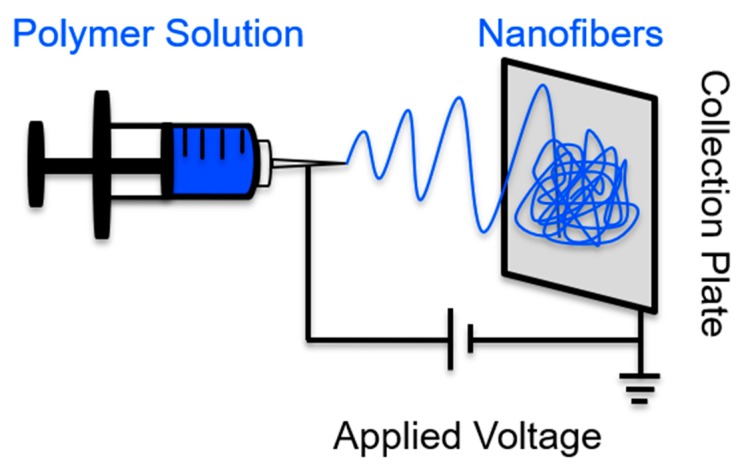
The cartoon displays the basic electrospinning process, including a high voltage supply and a syringe/needle that is held at a fixed distance from a collection plate. To control the polymer solution flow rate, an advancement pump is typically used. Adapted, with permission, from Meng et al. [35].

A basic, home-built electrospinning device only needs a few components, as displayed in Figure 1. A circuit is required to produce an electric field, which is created using electrodes that connect any sort of collection plate to a metal needle. A syringe is loaded with a precursor solution (polymer melt, solution, or sol gel) and is slowly pushed forward so that the surface tension can hold a pendent drop of the precursor solution in place at the edge of the needle. Coinciding with an increase in applied voltage, repulsive electrical forces are responsible for pulling a pendent drop of precursor solution into a Taylor Cone [32,33]. When the critical voltage is applied, the surface tension forces are overwhelmed by the electrical forces, causing a jet to exit the Taylor Cone. The polymer solution jet becomes unstable, thus causing bending, stretching, and whipping to occur. In less than 20 s, solid polymer fibers are deposited on the collection plate, because the bending instabilities coincide with the solvent evaporation [34]. Notably, viscosity optimization is one method of tailoring the morphology of the accumulated nanofibers within the mat. For example, if the precursor’s viscosity is too low, the Rayleigh instabilities will yield fibers that are not smooth and continuous, possibly forming beaded fibers.

Through polymer selection, nanofibers can be antibacterial or antifouling. Chitosan nanofibers intrinsically exhibit antibacterial activity [36], whereas hydrophilic polymers have been electrospun and could be considered for the basis of antifouling nanofiber mats, including poly(ethylene oxide) (PEO) and cellulose [37,38,39,40]. However, hydrophilicity alone is insufficient to produce long-lasting effective antifouling nanofibers. Through further alterations, nanofibers can exhibit more intense antibacterial or antifouling properties.

The fiber mats that are produced by electrospinning can easily be optimized during or after the fibers are produced for specific applications [41]. Tailoring the release rate of antimicrobial agents from a nanofiber mat can be accomplished by changing the precursor solution, because the polymer matrix in which the agents are embedded is often largely responsible for the mat’s degradation [42]. Alternatively, pharmaceuticals or active agents can be decorated on the exterior of the nanofibers [43]. Implementing solution blending [44,45,46,47], coaxial electrospinning [48,49,50,51,52,53], or emulsion electrospinning [54,55,56,57,58] allows for the encapsulation of active agents inside the fibers. Many different surface science techniques can be used to alter fabricated nanofiber mats, including electrostatically or covalently attaching functional moieties [59,60,61,62], dipping the produced mats into a polymer bath to apply a coating [63,64,65], or using self-assembly techniques [66]. Typically, the modification of nanofiber mats after the fibers are produced results in functional moieties that are located on the outside of the nanofibers. An in-depth discussion about selecting an appropriate precursor solution and how to optimize the inclusion of the functional species within nanofiber mats has been well documented in our previous review article [67].

## 3. Established and Emerging Approaches to Engineering Antibacterial and Antifouling Surfaces and Nanofiber Mats

The associated risk of infection has led to the interest of antibacterial and antifouling surfaces for applications including surgical products and wound healing patches [6,7,68,69]. For economic and functional reasons, electrospun nanofibers show great promise for use as antibacterial and/or antifouling materials, because of their nano-effects, including their great surface energy, chemical reactivity, and reported conductivity (i.e., thermal and electrical) [70]. Nanofiber mats can be used as a conformal surface coating or as a free-standing material to provide a controlled interaction with microorganisms. Nanofiber mats can coat surfaces and impart their antibacterial properties to the underlying substrate. For example, while hydrophilic cellulose nanofibers rapidly uptake viable microbes [29] by surface functionalizing the cellulose nanofibers with the cationic polymer poly(diallyldimethylammonium chloride) (pDADMAC), the mats were transformed into contact killing materials that inactivated ~97% of the *Escherichia coli* (*E. coli*) [63].

Because of their highly porous yet interconnected structure and high surface area, nanofibers are a great scaffold to deliver biocidal agents, able to protect wounds while administering desirable therapeutics [71]. For instance, polymers such as poly(vinyl pyrrolidone) and poly(dopamine methacrylamide-co-methyl methacrylate) have been electrospun so as to deliver iodine and silver nanoparticles, respectively, acting as topical drug delivery systems [71,72,73]. In the sections below, we will describe the general approaches to engineering materials to be either antibacterial or antifouling (Section 3.1 and Section 3.3), complimented by examples of nanofiber mats that are engineered to combat biofouling (Section 3.2, Section 3.4, and Section 3.5).

### 3.1. General Approaches to Engineering Antibacterial Surfaces

**Figure 2:**
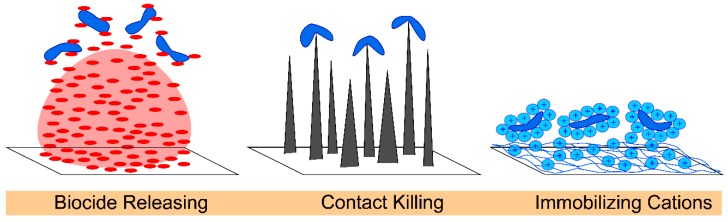
Antibacterial surfaces inactivate microorganisms by either releasing biocides, having mechano-bactericidal nanotopographies, or via immobilized cationic species.

Antibacterial surfaces are either bacteriostatic or bactericidal, often through an interference with bacterial DNA, protein synthesis, or the activity of enzymes that are involved with bacteria cell metabolism [74]. Antibacterial surfaces kill bacteria by releasing drugs, direct contact with cell membranes, or expressing cationic polymers, Figure 2. Biocide-releasing antibacterial surfaces commonly use silver, triclosan, or chitosan because of their effects on both Gram-positive and Gram-negative bacteria [36,75,76,77,78,79,80]. The challenges of using release biocides include the development of resistance, optimization of their release duration and kinetics, and maintenance of a high enough concentration to continually eliminate bacteria [5,15,81].

Materials that kill microorganisms via a contact-killing mechanism can inactivate bacteria through their surface topography. Lin et al. provides an excellent review on how nanomaterial geometry can cause bacterial cell death by delivering lethal mechanical forces [82]. Some examples include nanotubes, especially high aspect ratio single walled carbon nanotubes, which kill microbes on contact because of a mechano-bactericidal mechanism [83]. Surface coatings that mimic cicada wings or gecko skin stretch the bacteria cells until they rupture [5,82,84,85,86]. Alternatively, contact-killing topographies inactivate bacteria through strong adhesion between pillars and bacterial cells, which are exasperated by the shear forces that are generated by the efforts of the bacteria to remove themselves from the unfavorable topography [82]. While single walled carbon nanofibers have been embedded within nanofibers [44,87,88], the surface of nanofibers have yet to be decorated with sharp geometries that kill microbes on contact.

Another approach is functionalizing the surface of nanofibers to present cationic charges, such as cationic proteins (or polypeptides [89]), quaternary ammonium moieties, or ammonium salts, which inactivate Gram-positive and Gram-negative microbes through the disruption of bacterial cell membranes [90,91,92]. The nitrogen in the ammonium salts contains positive charges that cause bacteria cells to lose various physiological functions, including osmoregulation, through its interaction with negatively-charged acidic phospholipids in the cell membrane. Quaternary ammonium cations are effective against bacteria when the alkyl groups contain 4–18 carbons; greater effects on Gram-positive bacteria have been reported in alkyl chains containing 14–16 carbons, whereas a greater effect on the Gram-negative bacteria have been reported for alkyl chains containing 12–14 carbons [15]. Unfortunately, the use of quaternary ammonium cations causes reduced biocide effectiveness and increased resistance over time [93]. The high efficacy of the antibacterial agents, including biocides, cationic polymers, and surface topography, acts as a foundation for engineering the nanofibers to directly deliver these agents to where they are most needed.

### 3.2. Specific Examples of Antibacterial Nanofiber Mats

To effectively kill Gram-positive and Gram-negative bacteria we need to have many different biocidal nanofiber mats to choose from, because antibiotic-resistant bacteria strains continue to emerge. For this reason, numerous antibacterial agents (inorganic, metallic, and organic) have been incorporated into the electrospun mats. Through the precise tailoring of antibacterial agents to address specific concerns, ‘the bad’ microorganisms are targeted without contributing to antibiotic resistance.

Inorganic materials, specifically titania, have been incorporated within the nanofiber mats. When compared with the control scaffolds, the titania nanofibers that were doped with iron killed *E. coli* when they were photoactivated [94]. By incorporating titania into polyurethane fibers, *Pseudomonas aeruginosa* (*P. aeruginosa*) and *Staphylococcus aureus* (*S. aureus*) were strongly inactivated [95]. An additional study demonstrated that the titania mats [96] inhibited the *E. coli* and *S. aureus* growth when the zinc concentrations of 0.4 μg/mL and 1.6 μg/mL, respectively, were tested.

Traditionally, nanofiber mats have been fabricated with incorporated metals because they can serve as strong antimicrobials [59,97,98,99,100]. One of the most commonly used metals is silver, because it has a wide spectrum of killing efficacy and a low bacterial resistance. When considering using electrospun mats as exterior bandages, silver has been used because it can lower inflammation at a wound site while encouraging calcium and the subsequent epithelialization. To avoid silver cytotoxicity, a chemical reducing agent is needed, which can either be aqueous or organic in nature [100]. The polymer poly(vinyl alcohol) (PVA) can reduce silver nanoparticles and serves as a quick, facile, and inexpensive processing agent, especially in comparison to other organic reducing agents that have been historically implemented. Hence, Nguyen et al. used PVA as a basis for their blended nanofibers with encapsulated silver nanoparticles [99]. To ‘pull’ the silver nanoparticles towards the exterior edge of the nanofibers, a heat treatment was applied to the electrospun blends. In addition to reducing the silver after the nanofiber mats were electrospun, the reducing capability of the PVA to produce silver nanoparticles during the electrospinning of polymer nanofibers has also been demonstrated [101,102]. Instead of incorporating silver nanoparticles within nanofibers, Schiffman et al. [59] decorated the surface of polysulfone fiber mats with silver nanoparticles. After the polysulfone fibers were fabricated, oxygen plasma was introduced so as to enable electrostatic interactions between the negatively charged scaffolds and the positively charged polyethyleneimine capped silver nanoparticles. The polysulfone nanofibers that were decorated with silver nanoparticles killed the Gram-positive bacteria, *Bacillus anthracis* and *S. aureus,* as well as *E. coli*. While the silver nanoparticles can be effective antimicrobial agents, as is the case with other metals, silver may delay the healing of a wound because it has been reported to cause irritation and bind to the DNA, preventing replication.

The actual mechanism by which silver nanoparticles kill microbes is as a result of silver ions leaching from their surface. Therefore, we fabricated zeolites that, once immobilized on a nanofiber scaffold, could deliver individual silver ions [103]. In-house, we synthesized Linde Type A (LTA) ilver ion exchanged zeolites (6.0 μm) on the outer surface of the cellulose nanofibers. Additionally, smaller LTA zeolites (0.2 μm) and three-dimensionally ordered mesoporous-imprinted zeolites (0.5 μm) were attached to the mats after the base cellulose nanofibers were fabricated. As a function of time, the silver ion release profiles, and the antimicrobial properties of the three cellulose nanofiber mats that were decorated with silver ion containing zeolites, were quantified. Large zeolites containing silver ions that were decorated on the surface of the nanofiber mats killed 11 times more *E. coli* cells than the free zeolites that were not immobilized on the nanofiber mats. An advancement from this work is the demonstration that the immobilized metal ion exchanged zeolites can deliver silver ions using a conformal nanofiber mat. Notably, zeolite nanocrystals that are immobilized on nanofiber mats can be engineered to deliver other antibacterial agents, including nitric oxide, copper, and zinc [104,105,106,107].

Nanomaterials that are carbon-based have strong antimicrobial properties against microorganisms [108,109,110]. Out of all of the carbon-based materials, single walled carbon nanotubes possess the greatest cytotoxicity because of a mechano-killing mechanism [83,111]. Schiffman et al. blended the single walled carbon nanotubes within the electrospun polysulfone nanofiber mats to study whether the composites would have antibacterial activity [44]. Indeed, the flexible composite nanofiber mats killed more *E. coli* as the concentration of the single walled carbon nanotubes was increased (and well-dispersed) within the nanofiber mats. For example, while a 0.1 wt % loading of single walled carbon nanotubes inactivated only 18% of the *E. coli*, the highest loading of single walled carbon nanotubes that were tested (1.0 wt %) inactivated 76% of the *E. coli*. Notably, the killing occurred in 15 min or less, which was the limit of the fluorescence based toxicity assay. In 2015, blended fibers containing poly(lactide-*co*-glycolide) (PLGA) with chitosan were electrospun and then they were subsequently decorated with graphene oxide that was covered with silver nanoparticles. The surface functionalization was possibly due to a reaction that occurred between the carboxyl groups of the graphene oxide and the primary amine groups that were available on the nanofiber scaffolding [112]. The *S. aureus*, *E. coli*, and *Pseudomonas aeruginosa* inactivation occurred because of the graphene oxide/silver nanoparticle nanofibers.

Nature has also inspired the search for alternative antibacterial agents. By releasing the plant derived antibacterial agents, shikonin and alkannin, from polymer nanofibers, both *S. aureus* and *E. coli* were strongly inactivated [113,114]. The PLGA nanofibers were electrospun and served as a carrier for a protein synthesis inhibitor called fusidic acid, which was derived from fungus. The growth of bacteria was prevented by the nanofibers [115]. Lysostaphin is a cell lytic enzyme that explicitly targets *S. aureus*. When researchers immobilized lysostaphin on the surface of cellulose-based nanofiber mats, the materials cleaved the pentaglycine cross-bridges in the peptidoglycan layer of the cell walls of *S. aureus*, while also exhibiting a minimal toxicity towards the keratinocytes (cells that are important in the proliferative phase of wound healing) [116]. Cinnamaldehyde is derived from cinnamon bark [117]; the essential oil was incorporated into electrospun chitosan/PEO nanofibers via a Schiff base reaction that occurred on the amine group of chitosan [36]. After 180 min of incubation, the control mats, because of their chitosan content alone, inactivated *P. aeruginosa* (~50%). However, the nanofiber mats exhibited an enhanced inactivation of the opportunistic human pathogen (~80%) by loading 5.0 wt % of cinnamaldehyde into the chitosan-based scaffolds. In a following investigation [118], the effect of rheology of the precursor polymer solution (with or without hydrophobic oils) on the spinnability and morphology of electrospun nanofibers was investigated. The solutions containing PEO and chitosan with various molecular weights and degrees of acetylation, as well as with different loadings of cinnamaldehyde and hydrocinnamic alcohol, were prepared in order to determine how the oil incorporation altered the solution viscosity and chain entanglement concentration. It was determined that 1:3 and 1:6 were the maximum polymer:oil mass ratio that could be electrospun for chitosan/PEO:cinnamaldehyde and :hydrocinnamic alcohol, respectively. A greater chitosan degree of acetylation only increased the incorporation of hydrocinnamic alcohol. To date, a plethora of plant-derived active agents have been electrospun into multifunctional nanofiber mats, including aloe vera, baicalein, chamomile, grape seed, green tea, Tecomella undulata, *Azadirachta indica*, *Calendula officinalis*, *Centella asiatica, Garcinia mangostana, Grewia mollis*, *Indigofera aspalathoides*, *Memecylon edule*, and *Myristica andamanica*, as discussed in a recent review paper [119,120]. The electrospinning of numerous plant-derived extracts and the rheological studies that revealed important chain entanglement parameters together imply that additional hydrophobic molecules could be electrospun into multifunctional and smart electrospun nanofiber delivery vehicles.

### 3.3. General Approaches to Engineering Antifouling Surfaces

**Figure 3:**
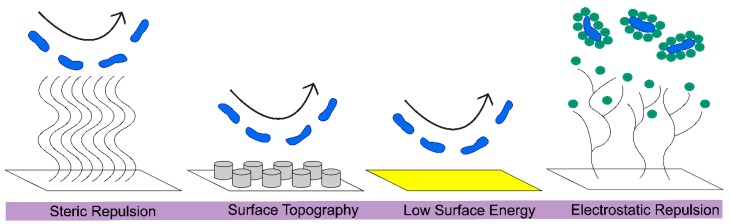
Antifouling surfaces repel microorganisms through steric repulsion, surface topography, low surface energy, or electrostatic repulsion.

Antifouling surfaces repel bacterial cells from attaching, often through unfavorable conditions that are manufactured on the engineered surface via the immobilization of polymers, such as PEG, or through the use of structured surfaces [19,121]. Overall, antifouling surfaces can be classified as using steric repulsion, surface topography, low surface energy, or electrostatic repulsion, to prevent bacterial attachment, Figure 3. Steric repulsion occurs because of the compression of long polymer chains that repel protein adsorption as a result of strong repulsive forces. Surface topography can create an adverse environment for bacterial attachment. For example, organized topography featuring square, rectangular, or circular posts significantly reduced the bacterial attachment [122], as previously reviewed [121]. Surfaces that are textured with highly aligned polystyrene nanofibers have been studied by Kargar et al. [123], so as to determine the effect that surface curvature, feature size, and spacing has on the adhesion of *P. aeruginosa.* A low surface energy prevents adsorption, while a high surface energy leads to adsorption and contaminated surfaces [124]. Electrostatic repulsion involves cationic polymers, causing cell lysis through the disruption of the negatively charged membrane of bacteria [15]. While currently it is more common to create nanofibers that incorporate antifouling polymers to repel microbes, in the future there will be opportunities to optimize the surface topography of nanofibers so as to solicit an antifouling effect, especially because the literature has established ways of changing the surface topography and the hierarchical organization of nanofiber mats [125].

### 3.4. Commonly Selected Polymers for Antifouling Nanofiber Mats

PEG, a biocompatible, hydrophilic polymer, has long been considered the ideal polymer for antifouling applications, due in part to the established and relatively easy methods of adding functional groups to PEG through developed means of conjugation [126]. PEG refers to polymers with a molecular weight that is less than 20 kDa, while poly(ethylene oxide) (PEO) denotes polymers with a greater molecular weight and the same chemistry. The covalent attachment of PEG to another molecule (PEGylation) adds hydrophilicity, stability, and antifouling properties to the molecule in question [16]. However, PEG, a non-biodegradable polyether that should not accumulate in the human body, sometimes leads to anaphylactic reactions by triggering complement activation [127]. Furthermore, PEG does not have long term stability, losing functionality over time because of oxidative degradation. The development of PEG antibodies and hypersensitivity reactions has raised concern for antifouling and antibacterial applications [127,128]. Because PEG auto-oxidizes to form aldehydes and ethers when it is exposed to oxygen in biological environments, inhibiting its ability to prevent protein adsorption, there is an increased interest in finding alternatives for antifouling applications [129].

Although PEG is the current gold standard for antifouling materials, the electrospinning of PEO/PEG nanofibers has been limited to PEG that has blended with additional polymers because of the lack stability of the all-PEG fibers [39,130,131,132]. Polymer zwitterions are another a class of antifouling materials that hold promise because of their chemical stability and fouling resistance, with comparable or improved cytotoxicity and antifouling properties in comparison with PEG [5,133,134,135,136]. These stable antifouling polymers pull water to the surface, creating a tight hydration barrier [137,138], which sterically and energetically prevents organic and biological matter from adsorbing [139]. The positive and negative charges of zwitterions allows for electrostatically induced hydration, while the PEG forms a hydration bubble through its hydroxyl groups. Thus, work on chemically and mechanically stable nanofibers with incorporated amphiphilic groups and polymer zwitterions will be discussed.

### 3.5. Specific Examples of Antifouling Nanofiber Mats

Surface chemistry, surface topography, and mechanical properties can all be tailored to delay the first adhesion of biomolecules and microorganisms on a surface [19,21]. Surfaces that are amphiphilic display areas on the length scale of a foulant, which are hydrophilic and hydrophobic, and have been demonstrated to be antifouling. The nanofibers that were prepared from amphiphilic triblock terpolymers and poly(lactic acid) exhibited a superior antifouling performance to the fibers without the amphiphilic groups [140].

Polymer zwitterions, an emerging class of antifouling materials, sterically hinder protein adsorption [141] and bacterial adhesion [142]. In 2009, the zwitterionic copolymers were electrospun using sulfobetaine methacrylate in a poly(*n*-butyl acrylate) matrix [143]. It was possible that the small fibers (100 nm) formed as a result of aggregations of the zwitterion groups, as opposed to reaching a critical chain entanglement, because the researchers reported that their precursor solutions had a low solution concentration and viscosity. Larger diameter fibers (200–800 nm) were electrospun when poly(sulfobetaine methacrylate) with a high molecular weight was employed in a high concentration precursor solution [144,145]. Multifunctional (antibacterial and antifouling) polymer zwitterion fibers containing silver were fabricated using a multi-stage process (polymerization, electrospinning, and photo-cross-linking) by Lalani and Liu [146]. Small diameter vascular grafts were created by electrospinning blends of polyurethanes with sulfobetaine groups; the polyurethanes provided a matrix that was elastic and biodegradable [147]. Recently, the nanofiber mats that have been electrospun from mixtures of poly(vinylidene fluoride) and random zwitterionic copolymers of poly(methyl methacrylate) and sulfobetaine-2-vinylpyridine (SB2VP) demonstrated a fivefold reduction in bovine serum albumin adsorption versus homopolymer poly(vinylidene fluoride) nanofibers [148]. Additionally, Ozcan et al. have electrospun the zwitterionic amphiphilic copolymer poly(trifluoroethyl methacrylate-random-sulfobetaine methacrylate) (PTFEMA-r-SBMA) into very hydrophobic (~140°) nanofibers that resisted the bovine serum albumin adsorption [149].

Presenting a polymer zwitterion on the outside of the fibers, instead of blending them within the fibers, would increase their likelihood to stop the protein and bacterial accumulation on nanofiber mats. Thus, to maximize the function of polymer zwitterions, our group has demonstrated a new technology that can decorate the surface of nanofibers with poly(2-methacryloyloxyethyl phosphorylcholine) (pMPC) [150]. Two scalable solution-based approaches, named sequential and codeposition, were demonstrated. For the sequential deposition method, dopamine was first polymerized on the nanofiber mats before the pMPC was physioadsorbed. Whereas in the codeposition method, the fibers were submerged in a bath that contained pMPC and dopamine. Statistically, both of the methods resulted in chemically stable surface modified fibers with the same diameter, contact angle measurements, and chemistry, which was confirmed using x-ray photoelectron spectroscopy. However, the nanofibers that were functionalized sequentially with polymer zwitterions had a rougher surface topography, which potentially had a strong influence on the observed differences in the materials’ ability to resist the attachment of proteins and microorganisms. The polymer zwitterion enhanced nanofibers that were produced using the sequential method exhibited an impressive flux recovery ratio of 95%, which was 300% better than the cellulose nanofibers, and were conducted after the materials were exposed to bovine serum albumin for 21 days. In terms of resisting bacterial attachment, after 24 h, both *E. coli* and *S. aureus* barely accumulated on either of the polymer zwitterion surface-modified nanofiber mats, a statistically significant difference than the control cellulose nanofiber mats. While the cellulose nanofiber mats are chemically and mechanically robust, by coating the high surface area materials with pMPC, we have created high performance antifouling materials that are ideal for use in tissue engineering applications and water purification technologies. The high efficacy of polymer zwitterions and amphiphilic groups as antifouling materials illustrates the continued need for fundamental research, so as to explore more economical and translatable means of developing antifouling surface coatings that delay biofouling for as long as possible.

## 4. Perspective and Conclusions

Biological fouling and antibiotic-resistance are complex problems, affecting a wide range of industries and necessitating multifaceted, engineered solutions. Traditional means of combatting detrimental bacteria using broad-spectrum antibiotics overlook the inherent complexities that lead to their decreased efficacy and the development of antibiotic-resistance. Consequently, fundamental research is needed to explore alternatives, namely more-targeted antibacterial agents, new antifouling surface coatings, and multifaceted approaches. Electrospinning presents a scalable platform that can be used to fabricate conformal coatings with inherent antibacterial/antifouling properties, or engineered to incorporate targeted antibacterial and antifouling agents that restrict biofilm formation. While electrospun polymer fibers are commercially viable [151], when industrially producing antibacterial electrospun nanofiber mats, we must aim to avoid the overuse of commercial antibiotic agents. To reduce the need for antibacterial agents, antifouling nanofibers should first repel microbes and proteins for as long as possible so as to delay their attachment. While we have shown that the ultrafiltration membranes that are surface modified with randomly accumulated nanofiber mats can delay biofouling [40], further studies that show the influence of the nanofiber surface topography on bacteria adhesion are needed [152]. Electrospun nanofiber mats will keep having a vital role in controlling the local interactions with microorganisms across medical, environmental, and diagnostic applications, because of the growing appreciation of convergent collaborations involving scientists and engineers from materials science, chemistry, microbiology, and medicine.
